# Lightning-Rod Effect of Plasmonic Field Enhancement on Hydrogen-Absorbing Transition Metals

**DOI:** 10.3390/nano9091235

**Published:** 2019-08-30

**Authors:** Norihiko Fukuoka, Katsuaki Tanabe

**Affiliations:** Department of Chemical Engineering, Kyoto University, Kyoto 615-8510, Japan

**Keywords:** transition metal, surface plasmon, nanoparticle, nanophotonics, hydrogen storage, sensing, nuclear fusion, energy device

## Abstract

The plasmonic enhancement of electromagnetic field energy density at the sharp tips of nanoparticles or nanoscale surface roughnesses of hydrogen-absorbing transition metals, Pd, Ti, and Ni, is quantitatively investigated. A large degree of energy focusing is observed for these transition metals in the microwave region, even surpassing the enhancement for noble metals according to the conditions. Pd, for instance, exhibits peak field enhancement factors of 6000 and 2 × 10^8^ in air for morphological aspect ratios of 10 and 100, respectively. Metal surfaces possibly contain such degrees of nano- or micro-scale native random roughnesses, and, therefore, the field enhancement effect may have been unknowingly produced in existing electrical and optical systems. In addition, for future devices under development, particularly in hydrogen-related applications, it is desirable to design and optimize the systems, including the choice of materials, structures, and operating conditions, by accounting for the plasmonic local energy enhancement effect around the metal surfaces.

## 1. Introduction

Free electrons in metallic materials, particularly around metal surfaces or interfaces with dielectric media, exhibit a strong interaction with electromagnetic fields or light in the form of collective oscillation, named “surface plasmons” [1,2,3]. Surface-plasmon-induced electromagnetic field enhancement on metal surfaces [4,5,6,7] has been utilized for various applications, such as chemical/biomedical sensing [8,9,10], photodetectors [11,12,13], light-emitting diodes [14,15,16], nanolasers [17,18,19], solar cells [16,20,21,22], and optical cloaking [23,24,25]. Recently, we found through numerical analysis that a large degree of energy focusing, with enhancement factors over several hundred, is available for planar surfaces of hydrogen-absorbing transition metals, Pd, Ti, and Ni, in the microwave region, even surpassing the enhancement for noble metals [26]. We therein discussed the potential applications of the plasmonic field enhancement effect on hydrogen-absorbing transition metals, such as hydrogen storage [27,28,29,30,31], sensing [32,33,34], and nuclear fusion [35,36,37,38]. In contrast to the studied plasmonic field enhancement effect on planar metal surfaces, it is known that surfaces with sharp curvatures allow the electromagnetic field to concentrate further, called the “lightning-rod effect” [39,40,41,42,43]. In the present study, we numerically investigate the plasmonic field enhancement on sharp surfaces of hydrogen-absorbing transition metals, Pd, Ti, and Ni.

## 2. Theory and Calculation Methods 

**Figure 1:**
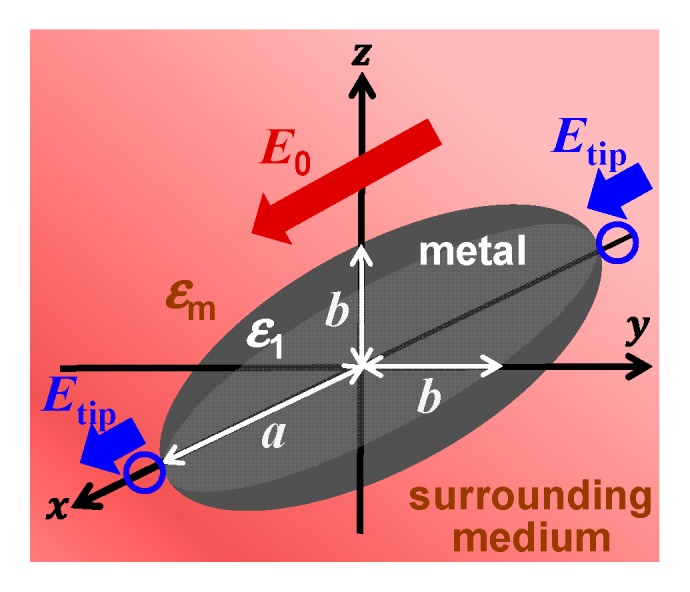
Schematic bird’s-eye view of the system considered in this study for the calculations of field enhancement factors.

We calculate the field enhancement factors, which represent the intensity ratios for fields around the object (metals in this case) to those in the absence of the object, or the original incident fields, for prolate-spheroidal metal nanoparticles in air, H_2_, or vacuum, and H_2_O. We specifically calculate the field enhancement factors at the tips of the prolate-spheroidal metal nanoparticles, to represent sharp-curvature metal surfaces. These calculations, based on the classical electromagnetic field theory in the quasistatic limit [7,44], quantitatively show how much energy can be concentrated from the incident optical or electric power. The intensities of electromagnetic fields around subwavelength-size metal nanoparticles can be described by the formalism below in the quasistatic limit [44]. Consider a homogeneous, prolate spheroid with radii of the major and minor axes *a* and *b*, respectively, placed in a medium in which there exists a uniform static electric field ${\vec{E}}_{0}$ applied along the major axis of the spheroid, as schematically depicted in Figure 1. If the permittivities or dielectric constants of the spheroid and the medium are different, a charge will be induced on the surface of the spheroid. The initially uniform field will, therefore, be distorted by the introduction of the spheroid. Based on the calculation schemes described in earlier articles [7,42,44,45], in short, the electric field outside the spheroid and at the tip of the prolate spheroid is given by

(1) $${\vec{E}}_{tip}=\frac{\varepsilon_{1}\left(\lambda \right)}{\varepsilon_{m}\left(\lambda \right)+L_{1}\left\{\varepsilon_{1}\left(\lambda \right)-\varepsilon_{m}\left(\lambda \right)\right\}}{\vec{E}}_{0},$$

where *ε*_1_ and *ε_m_* are the frequency-dependent complex permittivities or dielectric functions of the spheroid and the surrounding medium, respectively. *L*_1_ is the geometrical factor for the major axis of the prolate spheroid, calculated as

(2) $$L_{1}=\frac{1-e^{2}}{e^{2}}\left(-1+\frac{1}{2e}\ln\frac{1+e}{1-e}\right),$$

where *e* is the eccentricity of the particle shape:

(3) $$e=\sqrt{1-\frac{b^{2}}{a^{2}}}.$$

The field enhancement factor is then calculated as

(4) $$\eta \equiv \frac{{\left|{\vec{E}}_{tip}\right|}^{2}}{{\left|{\vec{E}}_{0}\right|}^{2}}={\left|\frac{\varepsilon_{1}\left(\lambda \right)}{\varepsilon_{m}\left(\lambda \right)+L_{1}\left\{\varepsilon_{1}\left(\lambda \right)-\varepsilon_{m}\left(\lambda \right)\right\}}\right|}^{2}.$$

Note that this field enhancement factor is defined as the ratio of field intensities and not field magnitudes. Incidentally, for the spherical case, which provides *L*_1_ = 1/3, *η* reduces to the equation of the field enhancement factor derived in Reference [7]. The empirical complex dielectric functions of metals and of the surroundings on the frequencies listed in References [7] and [26] are used for the computations in this study. We assume that *ε*′ = 1 and *ε*″ = 0 throughout the entire frequencies for air, H_2_, and vacuum. The electrostatic calculations carried out in this study are valid for particle sizes in the range of 10–100 nm, for which the phase retardation is negligible throughout the particle, the field enhancement will be largest, and metal nanoparticles and nanoscale roughnesses will, therefore, become most applicable, as discussed in the Results and Discussion section.

## 3. Results and Discussion

**Figure 2:**
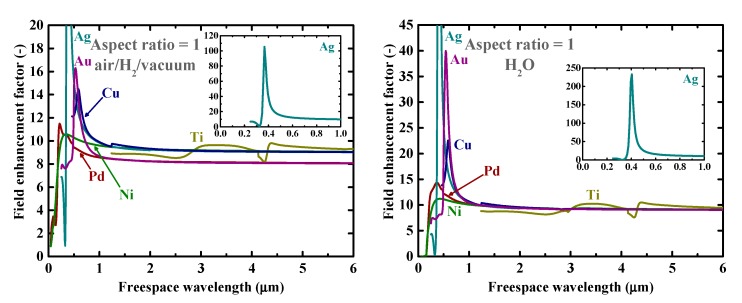
Calculated electromagnetic field enhancement factors around spherical nanoparticles of Au, Ag, Cu, Pd, Ti, and Ni in (**left**) air/H_2_/vacuum and (**right**) H_2_O. The insets are the clarified plots for Ag.

**Figure 3:**
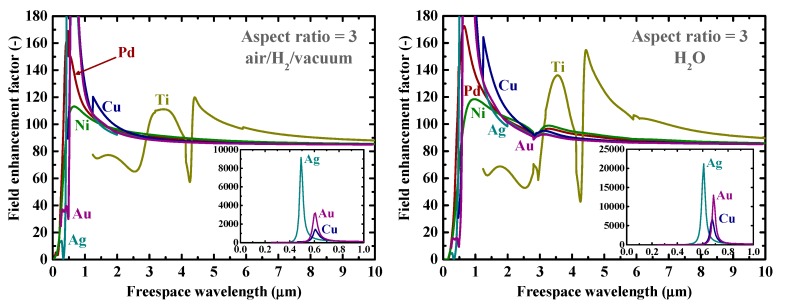
Calculated electromagnetic field enhancement factors at the tips of spheroidal nanoparticles of Au, Ag, Cu, Pd, Ti, and Ni with an aspect ratio of 3 in (**left**) air/H_2_/vacuum and (**right**) H_2_O. The insets are the clarified plots for Au, Ag, and Cu.

**Figure 4:**
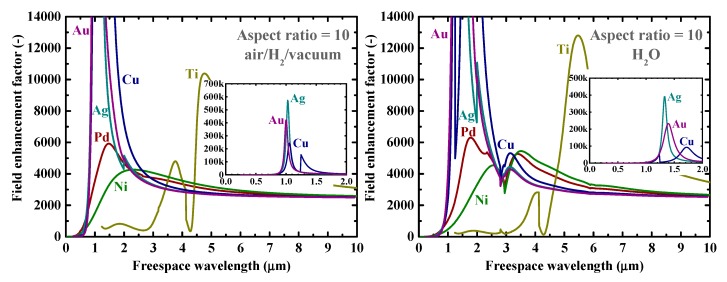
Calculated electromagnetic field enhancement factors at the tips of spheroidal nanoparticles of Au, Ag, Cu, Pd, Ti, and Ni with an aspect ratio of 10 in (**left**) air/H_2_/vacuum and (**right**) H_2_O. The insets are the clarified plots for Au, Ag, and Cu.

**Figure 5:**
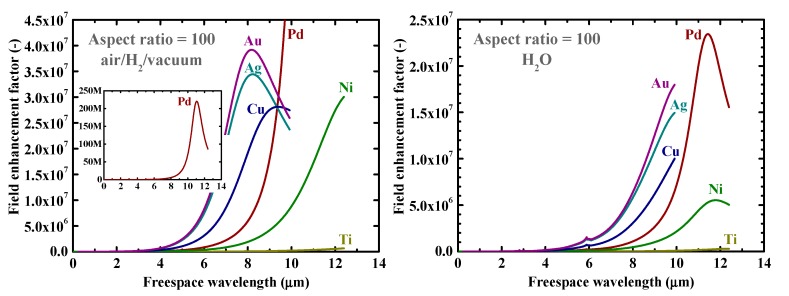
Calculated electromagnetic field enhancement factors at the tips of spheroidal nanoparticles of Au, Ag, Cu, Pd, Ti, and Ni with an aspect ratio of 100 in (**left**) air/H_2_/vacuum and (**right**) H_2_O. The inset of (**left**) is the clarified plot for Pd.

**Figure 6:**
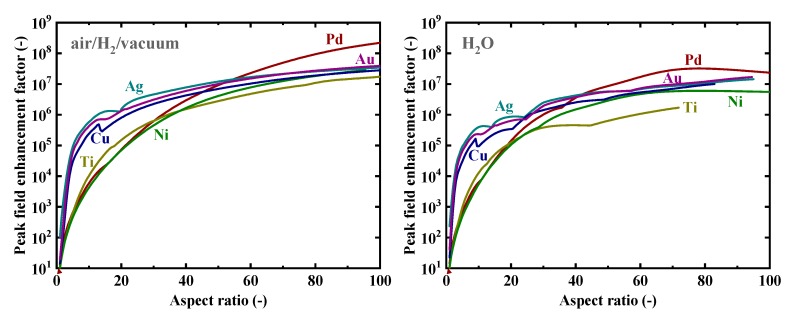
Dependence of the peak electromagnetic field enhancement factors at the tips of spheroidal nanoparticles of Au, Ag, Cu, Pd, Ti, and Ni on the aspect ratio in (**left**) air/H_2_/vacuum and (**right**) H_2_O.

**Figure 7:**
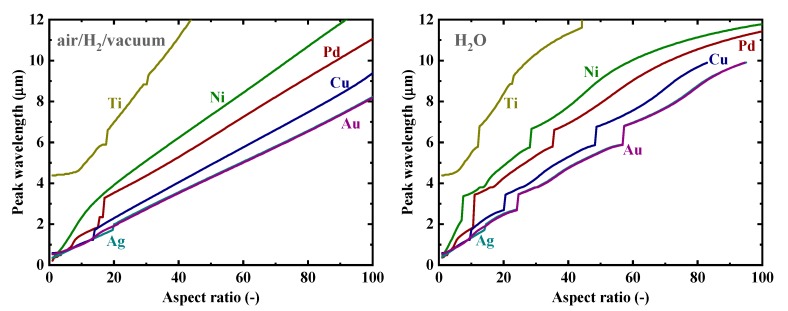
Dependence of the peak wavelength of the electromagnetic field enhancement factors at the tips of spheroidal nanoparticles of Au, Ag, Cu, Pd, Ti, and Ni on the aspect ratio in (**left**) air/H_2_/vacuum and (**right**) H_2_O.

Firstly, as a reference, we present in Figure 2 the calculated electromagnetic field enhancement factors for the simple, spherical nanoparticle case, which corresponds to the spheroid’s aspect ratio, *a*/*b*, of one. The peaks seen in these spectra are associated with the resonance or surface mode, characterized by internal electric fields with no radial nodes. A local energy enhancement around 10 times is decently observed for the hydrogen-absorbing transition metals, Pd, Ni, and Ti, in this spherical-shape case. Incidentally, the results in Figure 2 for shorter wavelengths are consistent with those reported in Reference [7]. It should be noted that the field enhancement factors, for our calculations, are independent of the particle size under the quasistatic approximation, and are valid for particle diameters around the range of 10–100 nm [7]. Figure 3 shows the field enhancement factors for spheroidal metal nanoparticles with an aspect ratio of three. It is observed that even for such a relatively small aspect ratio or morphological surface sharpness, enhancement factors over 100 are attainable for Pd, Ni, and Ti for a wide range of frequencies, through visible to infrared. Such nanoparticles or nanoscale surface roughness thus concentrate electromagnetic or optical energy in their vicinity like antennae. The artifact discontinuities for the curves for Cu and Ti around 1 and 4 μm, respectively, in Figure 3 and Figure 4 are incidentally because of the discontinuities in the source empirical data of the dielectric functions. Remarkably, electromagnetic field enhancement factors of several thousand are observed for Pd, Ni, and Ti for the aspect ratio of 10 (Figure 4). The resonant peak enhancement factors for Au, Ag, and Cu are observed to be even larger, on the order of 10^5^. Among the whole metal elements, Al and the noble metals Ag, Au, and Cu are known to exhibit distinctively higher field enhancement factors than other metals because of their high electrical conductivities [7,46]. Therefore, the combination of such noble-metal spheroidal nanoparticles and hydrogen-absorbed transition metals, available, for instance, by coating bulk metal surfaces by colloidal metal nanoparticles, may be another strategy for applications to harvest the photonic or electromagnetic energy focusing effect. Strikingly, as observed in Figure 5, the field enhancement factor of Pd for the sharp particle or surface morphology case of an aspect ratio of 100 reaches the order of 10^8^ in the infrared region, even exceeding those for the noble metals. This consequence is consistent with the results reported in Reference [26] that the plasmonic field enhancement factors of the hydrogen-absorbing transition metals become higher than those of noble metals for planar metal surfaces. Incidentally, the peak or resonant wavelength of Ti may unfortunately locate outside of the range of frequencies handled in this study. Figure 6 and Figure 7 summarize the dependence of the peak field enhancement factors and wavelengths, respectively, on the aspect ratio. It is observed that the electromagnetic field enhancement factors dramatically increase with the aspect ratio, namely, with the sharpness of the metal surfaces. As the sharpness increases, the resonant peak wavelength red-shifts. Large-aspect-ratio metal particles or high-curvature edges of surface irregularities exhibit high polarizabilities and, thus, large dipole moments, particularly at the resonance, to produce strong local field enhancement in the vicinity of such edges [44,47]. To discuss the electromagnetic similarity between isolated metal spheroidal particles and rough surfaces, it is worth mentioning that the detailed numerical calculation results reported in Reference [40], where rough metal surfaces were modeled as prolate hemispheroids protruding from a grounded flat plane, are quantitatively similar to our results in Figure 6 and Figure 7, for the peak field enhancement factors and wavelengths.

The large field-enhancement effect on the hydrogen-absorbing transition metals, Pd, Ni, and Ti, observed in the series of calculations in this study can be used for various hydrogen-energy applications. As discussed in Reference [26], potential applications include hydrogen storage, sensing [48,49], laser fusion [46], and condensed-matter fusion. In addition, for the reported experiments so far, for instance, in the condensed-matter nuclear fusion field, it is highly possible that the deuterium-absorbed Pd, Ni, and Ti surfaces contained certain degrees of nano- or micro-scale native random roughnesses [39,40,50,51] corresponding to such morphological aspect ratios as those studied in this article. Therefore, some of the experimental material systems may have unknowingly benefited from the plasmonic field enhancement effect. The electrostatic calculation results shown in this paper are valid for particle sizes smaller than the fields’ wavelengths at which the phase retardation is negligible throughout the particle object. In addition, the dielectric functions of materials used for our calculations are empirical values for bulk materials, whose validity is debatable when the particle sizes become smaller than 10 nm, because of the electron mean free path limitation or scattering of conduction electrons off particle surfaces [44,47,52,53]. The calculation results for optical wavelengths under the quasistatic approximation are, therefore, valid for metal particles with diameters in the range of 10–100 nm. Metal particles with sizes smaller and larger than these limits both exhibit broader plasmon resonances and smaller field enhancements, because of the surface scattering losses and the radiative losses or electrodynamic damping, respectively [47,52,53]. Therefore, the choice of particle sizes, 10–100 nm, for our calculations is most suitable for plasmon-enhanced electromagnetic and optical applications, because of the largest field enhancements. This size aspect should, therefore, also be accounted for in the optimized design of the material structures in potential applications. In addition, surface plasmons located in between multiple metallic objects with nanoscale separation distances, or so-called “gap plasmons” [54,55,56], would also provide large field enhancements on the conditions. The gap-plasmon effect for hydrogen-energy applications is important partially because gap plasmons are also commonly observed in real structures such as rough metal surfaces, and will be discussed in future work.

## 4. Conclusions

In this work, we numerically investigated the lightning-rod effect of plasmonic field enhancement on hydrogen-absorbing transition metals. A large degree of energy focusing was observed for these transition metals in the microwave region, even surpassing the enhancement for noble metals according to the conditions. Pd, for instance, exhibited peak field enhancement factors of 6000 and 2 × 10^8^ in air for morphological aspect ratios of 10 and 100, respectively. The metal surfaces possibly contained such degrees of nano- or micro-scale native random roughnesses, and, therefore, the field enhancement effect may have been unknowingly produced in existing electrical and optical systems. Active utilization of the plasmonic local energy enhancement effect around the metal surfaces by proper material and structure choices, such as the introduction of sharp nanoparticles or sharply roughened surfaces, can potentially improve hydrogen-related device performance.
